# Panoramic and skull imaging may aid in the identification of multiple myeloma lesions

**DOI:** 10.4317/medoral.22123

**Published:** 2017-12-24

**Authors:** Karina-Morais Faria, Thais-Bianca Brandão, Wagner-Gomes Silva, Juliana Pereira, Frederico-Sampaio Neves, Marcelo-Corrêa Alves, Werner-Harumiti Shintaku, Marcio-Ajudarte Lopes, Carolina-Prado Ribeiro, Cesar-Augusto Migliorati, Alan-Roger Santos-Silva

**Affiliations:** 1 Oral Diagnosis Department, Semiology Area, Piracicaba Dental School, University of Campinas (UNICAMP), Piracicaba, São Paulo, Brazil. Av. Limeira, 901, Areão, Piracicaba, São Paulo, Brazil, CEP: 13414-903; 2 Dental Oncology Service, Instituto do Câncer do Estado de São Paulo [ICESP], Faculdade de Medicina da Universidade de São Paulo, São Paulo, Brazil. Av. Dr. Arnaldo, 251, Cerqueira César, São Paulo, Brazil, CEP: 01246-000; 3 Hematology Service, Instituto do Câncer do Estado de São Paulo [ICESP], Faculdade de Medicina da Universidade de São Paulo, São Paulo, Brazil. Av. Dr. Arnaldo, 251, Cerqueira César, São Paulo, Brazil, CEP: 01246-000; 4 Department of Propedeutics and Integrated Clinic, Division of Oral Radiology, Federal University of Bahia, Salvador, BA, Brazil; 5 Systems Analyst of the Technical Section of Informatics at Luiz de Queiroz College of Agriculture (ESALQ), University of São Paulo, Piracicaba, São Paulo, Brazil. Av. Pádua Dias, 11, São Dimas, Piracicaba, São Paulo, Brazil, CEP: 13418-900; 6 Department of Diagnostic Sciences and Oral Medicine, University of Tennessee Health Science Center (UTHSC) College of Dentistry, Memphis, Tennessee, United States. Union Avenue, 875, Memphis, Tennessee, United States, zipcode 38103; 7 Shared senior authors

## Abstract

**Background:**

The purpose of this study was to investigate the presence of punched-out lesions in craniofacial bones using three different radiographic protocols in a large cohort of patients.

**Material and Methods:**

One hundred fifty-five MM patients were evaluated using panoramic and skull (frontal and lateral) radiographs, which were performed in all patients at the time of MM diagnosis. The diagnostic potential for detecting punched-out lesions was compared among the radiographic techniques.

**Results:**

MM punched-out lesions were identified in 135 (87%) panoramic radiographs, 141 (91%) frontal and 144 (93%) lateral skull radiographs. Punched out-lesions were synchronously present in skull and jawbones in 129 (83.23 %) cases. The lesions were detected exclusively in skull in 18 (11.61%) cases and exclusively in jawbones in 6 (3.87%) cases. Punched out-lesion mainly affected the skull and the jawbones in a synchronous way (*p*<0.001) rather than separately.

**Conclusions:**

All investigated radiographic techniques (panoramic, frontal and lateral skull approaches) demonstrated high detection rates for MM punched-out lesions in craniofacial bones. Panoramic radiography may aid to the radiographic protocols to identify multiple myeloma bone lesions.

** Key words:**Multiple myeloma, osteolytic lesions, panoramic radiography.

## Introduction

Multiple myeloma (MM) is a hematological malignant neoplasm originated from plasma cells, which results in the production of abnormal antibodies known as “M proteins” and widespread bone damage (1). One of the challenges in the MM diagnosis is the need for a clinicopathological and radiological correlation; including the identification of bone lesions, hypercalcemia and renal failure, among others (2).

According to The International Myeloma Working Group (IMWG), one or more osteolytic lesions have to be visualized on skeletal radiograph for the diagnosis of MM.

In this context, the IMWG recommends performing conventional radiographs for the differentiation of MM from other monoclonal plasma cell diseases (3,4). In addition, the Durie-Salmon-Staging (4) MM system considers the presence and the number of osseous lesions identified on radiographs to predict the prognosis of the disease (5).

The classic MM bone lesion visualized in radiographs is a sharply defined and small lytic lesion with the so-called “punched-out” appearance (1). Single or multiple well-defined punched-out radiolucencies often present as the first signal of MM. Nearly 80% of all newly diagnosed cases of MM reveal these bone changes in conventional radiographies (5,6). The following sites are most commonly affected in MM patients: vertebrae (65%), ribs (45%), skull (40%), shoulders (40%), pelvis (30%), long bones (25%) and jawbones (20% to 30%) (1,2,5,6).

The IMWG recommends for each newly diagnosed patient with MM a complete conventional radiograph status, including skull, cervical, thoracic and lumbar spine (frontal and lateral views), chest (frontal view), pelvis (anterior-posterior view) and long proximal bones (anterior-posterior view) (1), additional views of any symptomatic area should also be acquired (2). In early stage disease, the role of radiographs is limited since approximately 50% of bone destruction due to MM occurs before there is any detectable radiographic alteration (5,7).

MM often occur in craniofacial bones as a primary manifestation. Bruce and Royer (1953) (8) and Miller *et al.* (9) reported that 20% to 30% of MM cases showed radiographic involvement of the jawbones. Symptoms associated with jawbones involvement in MM are uncommon whereas the most frequent clinical manifestations of this disease in the jawbones are paresthesia, pain, swelling and tooth mobility (10,11). In some cases, jawbones involvement presenting punched-out lesions may occur with a similar presentation to other cysts and odontogenic lesions (11). The osteolytic lesions are more common in the mandible than maxilla, especially in posterior teeth region, ramus, and condyle, probably due to the increased hematopoietic activity in these areas (9).

In this scenario, the identification of the MM manifestations in craniofacial bones is necessary to avoid delays in diagnosis as well as for the evaluation of the response to systemic treatment (12-15). Therefore, this study tested the hypothesis if the panoramic radiographic may considered a useful tool to detect osteolytic lesions of the maxillofacial complex of MM patients.

## Material and Methods

-Patients and study design

The present study was a collaboration among the University of Campinas, Piracicaba Dental School, Brazil; the Dental Oncology Service of the Instituto do Câncer do Estado de Sao Paulo (ICESP), Brazil and the University of Tennessee Health Science Center, College of Dentistry in Memphis (UTHSC-CD), United States. This study was approved by the Ethics Committee of the University of Campinas (protocol 118/2014) and the Institutional Review Board of The University of Tennessee Health Science Center-UTHSC (number 516827). This was a cross-sectional retrospective study performed with individuals treated at the Hematology Service of Instituto do Câncer do Estado de São Paulo from April/2010 to June/2014.

One hundred fifty-five patients diagnosed with MM were included in this retrospective study. Criteria for patients inclusion were: 1) a confirmed diagnosis of MM presenting with bone disease after complete clinical workup according to IMWG (2,3); 2) a digital panoramic radiograph obtained upon diagnosis; 3) skull radiographs (anterior and lateral approaches); 4) complete medical records. The exclusion criteria were the presence of non-MM neoplastic bone disease or absence of radiographs (panoramic, anterior and lateral skull). The Durie-Salmon (4) staging default method was used for the clinical staging of MM.

To assess the involvement of craniofacial bones, a descriptive approach was performed in 155 frontal and 155 lateral radiographs of the skull. In addition, digital panoramic radiographs (n=155) were analyzed for each patient involved in this study. The electronic records were consulted to access for information about the occurrence of skeletal complications.

-Radiographic evaluation 

All panoramic radiographs were taken in a dental X-ray machine (PaX- 400, Hawseongsi, Gyeonggido, Korea), using 68 kVp, 8 mA with an exposure time of 14s. All skull radiographs were taken in X-ray machine (OPTILIX 150/30/50 HC-100; Siemens, focal spot 0.6/1.0 mm), using 65 kVp, 10 mA and exposure time of 125ms. The radiographs were coded to protect health information. Radiographic images were independently evaluated at the UTHSC-CD by a radiologist certified by the American Board of Oral and Maxillofacial Radiology and an Oral Medicine practitioner certified by the American Board of Oral Medicine, images were displayed on a 24 inch LCD flat panel display (UltraSharp 2408WFP, Dell Inc., USA) with a screen resolution of 1920 x 1200 pixels in a room with reduced light.

Digital panoramic, lateral and frontal radiographs were evaluated separately. For identification of the presence of the osteolytic lesions, Radiographs were classified as score “present” (1) (when osteolytic lesions were evident) or “absent” (0) (no osteolytic lesions). All anatomical structures in the maxillo-mandibular complex were included in the radiographic evaluation. The observers were blinded to clinical data. In order to avoid inter-examiner variability in interpretation of the images, the observers performed all assessments under dim light conditions, without brightness and contrast adjustment. Interexaminer agreements were assessed using Cohen’s Kappa test to analyze the reliability of the examiners and the agreement was considered fair if Kappa was between 0,20-0,40, moderate if Kappa was between 0,40-0,60 and substantial if Kappa was between 0,60-0,80.16

-Data analysis

To verify the presence of punched-out lesions affecting skull and the jawbones the chi-square test of likelihood ratio was applied to test the capacity of diagnostic for both radiographic techniques (digital panoramic and skull Radiographs). The significance level of 5% was adopted and the analyses were performed through the system SAS (Institute Inc. The SAS System, release 9.3. SAS Institute Inc., Cary: NC.2010).

## Results

Clinicopathological data of studied patients are described in  Table 1. Bone complications status is described in  Table 2. Interexaminer Kappa test was 0.7916 and was considered appropriate for this study. Sixty-eight (43.8%) patients received intravenous bisphosphonate therapy for bone disease control. In terms of comorbidities, 63 (41%) patients reported hypertension, 26 (17%) heart conditions, 18 (12%) diabetes mellitus, 17 (11%) renal insufficiency, 4 (3%) hyperparathyroidism and 3 (2%) hypothyroidism.

**Table 1:**
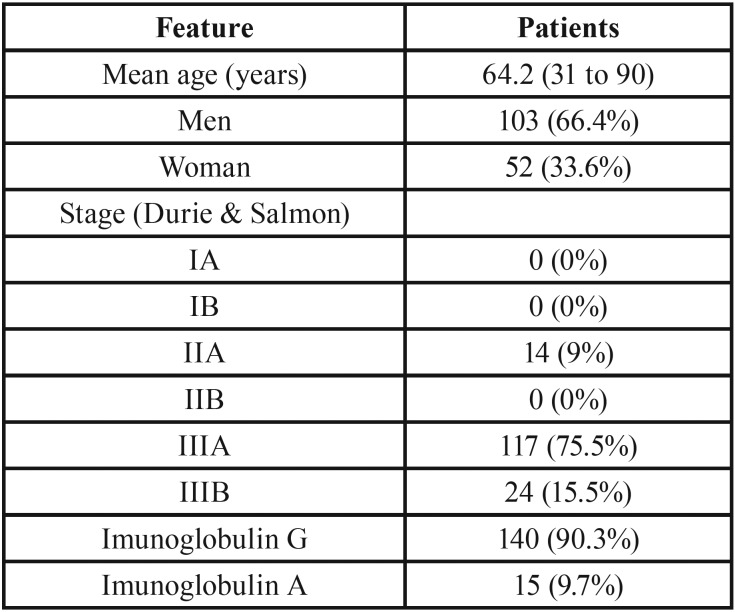
Clinicopathological features of studied multiple myeloma patients.

**Table 2:**
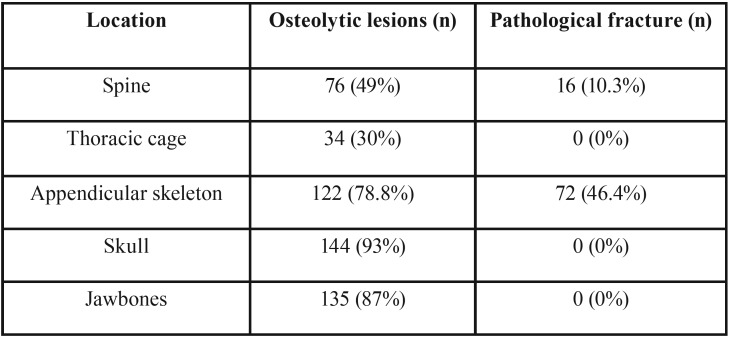
Bone complication status.

-Radiographic findings 

**Figure 1:**
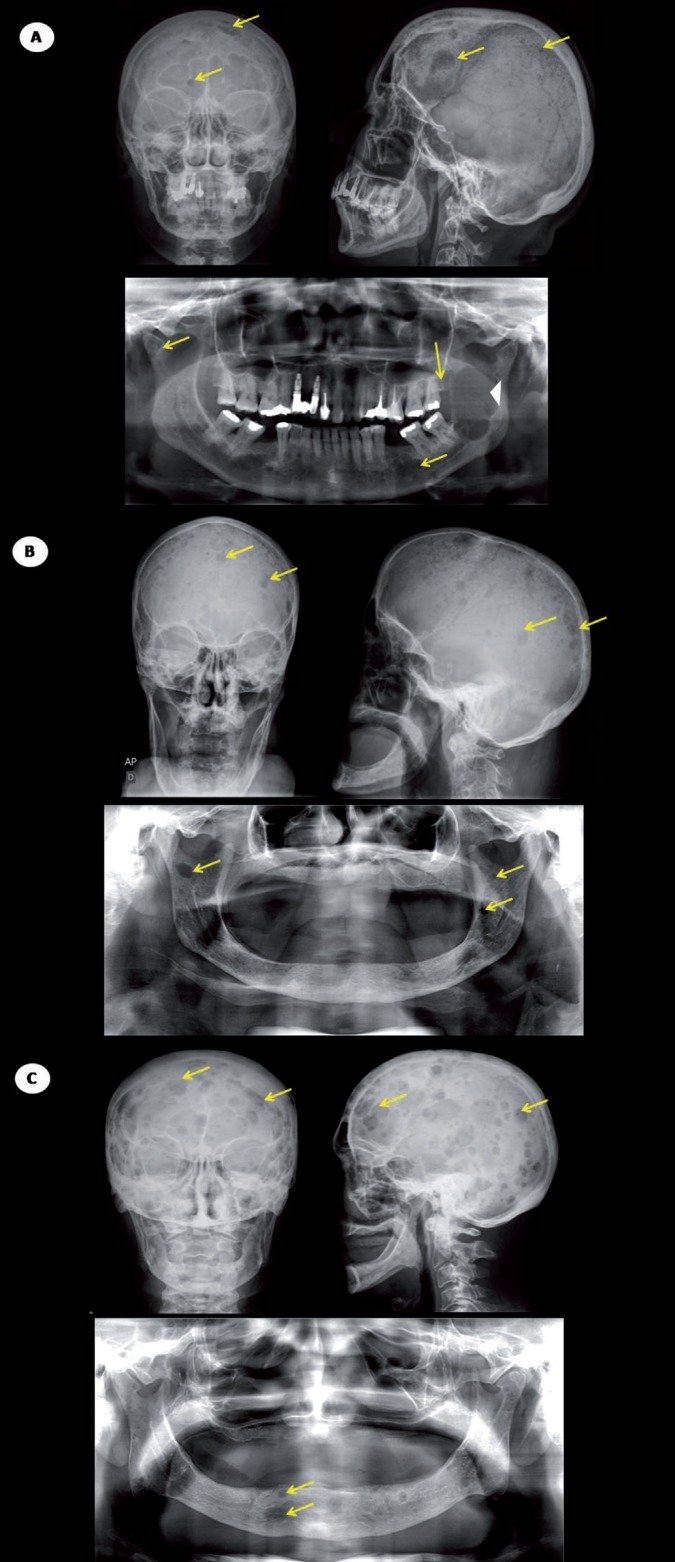
A. Digital panoramic radiography showing punched-out lesions (arrows) affecting maxilla and mandible and a large osteolytic image (arrowhead) affecting the left ramus of the mandible. Frontal and lateral skull radiographs of the same patient presenting multiple punched-out lesions (arrow). B. Digital panoramic radiographic evaluation showing punched-out lesions (arrows) affecting maxilla and mandible. Frontal and lateral skull radiographs of the same patient presenting multiple punched-out lesions (arrow). C. Digital panoramic radiographic evaluation showing punched-out lesions (arrows) affecting maxilla and mandible. Frontal and lateral skull radiographs of the same patient presenting multiple punched-out lesions (arrow).

A total of 135 (87%) patients presented punched-out lesions on the jawbones detected on panoramic radiographs, 141 (91%) patients presented punched-out lesions in frontal skull radiographs and 144 (93%) patients presented punched-out lesions in lateral skull radiographs. All punched-out osteolytic lesions in the skull were observed in frontal, parietal and occipital bone (Fig. 1A-C). Punched out-lesions were synchronously present in the skull and jawbones in 129 (83.23 %) of the cases, detected exclusively in the skull in 18 (11.61%) cases and exclusively in jawbones in 6 (3.87%) cases. The chi-square test revealed that punched out-lesions mainly affected the skull and the jawbones in a synchronous way (*p*<0.001) than separately.

When jawbones were independently evaluated, it was possible to observe that punched-out lesions affected the mandible in 135 (87%) patients and maxilla in 20 (13%) patients. When skull bones were independently evaluated, it was possible to observe that punched-out lesions affected the parietal bones in 139 (89.6%) patients, the frontal bone in 113 (72.9%) patients and occipital bone in 72 (46.4%). All punched-out lesions identified in the patients of this study were characterized as well-defined osteolytic lesions with variable size.

## Discussion

MM is a devastating malignancy of antibody-producing plasma cells that extensively affects the bone marrow. There is a slight male predominance. The median age at onset is 66 years, and only 2% of patients are younger than 40 years of age at diagnosis (17). The clinicopathological profile of the patients evaluated in the present study is in accordance with previous reports for clinical aspects of myeloma. The cohort of patients investigated in this study presented advanced stage MM with diffuse skeletal complications in the spine, thoracic cage and appendicular skeleton.

Bone fractures are an important health care concern among MM patients with advanced disease because they can interfere with functional independence and shorten survival. Approximately 45% of patients with MM experience a fracture in the first year after diagnosis (18). In accordance to the literature, our study presented a high number of patients with fractures, which were mainly identified in the appendicular skeleton.

Jawbones osteolytic lesions are not usually an isolated radiographic finding in MM patients, they are often observed synchronously to lesions on the skull and other bones (19-22). There are only a few available studies that have previously described the radiographic manifestations of MM in craniofacial bones, most of which represent small case series or isolated case reports (10-12,19,20). Futurani *et al.* (21) published the only study that analyzed both jawbones and skull involvement in a series of 38 MM patients using non-digital radiographs; they found 5 (13%) patients with mandible osteolytic lesions, no lesions in the maxilla and 5 (13%) patients with skull osteolytic lesions. The current study was the first to use digital panoramic and skull radiographs to investigate MM patients. Possibly, this methodological approach explains why this seems to be the first study to report the presence of well-defined MM osteolytic lesions in the maxilla as well as the highest rates of craniofacial bones involvement by MM osteolytic lesions (mandible: 87%, maxilla: 13%; parietal bones: 89.6%; frontal bone: 72.9% and occipital bone: 46.4%).

Panoramic radiographic is a routine exam and it is readily accessible to dental health care professionals that present low costs if compared with medical computed tomography (medical CT) (6,23-26). The traditional standard imaging technique for evaluation of bone disease in MM is the skeletal survey and there is no evidence in the literature that panoramic radiographic is included on protocols of radiographic evaluation for MM (1,5,6). Conversely, the IMWG recommendation to identify bone lesions include other imaging techniques, such as MRI, medical CT, whole-body low-dose computed tomography, WBLDCT, and FDG PET-CT (23,25), however, these sensitive techniques depends on availability and access.

Interestingly, the present study originally described a high incidence of osteolytic punched-out lesions observed in digital panoramic radiographs and skull radiographs (frontal and lateral) in MM patients, suggesting that digital panoramic radiographs are useful tools for a correct evaluation of the extent of disease in craniofacial structures – in addition to frontal and lateral views of the skull imaging technique, since MM manifestations may occur exclusively (mainly at early stages) on the jawbones (2,3,10,11). Furthermore, most of the studied patients presented the punched out-lesions affecting the skull and the jawbones in a synchronous way. However, to date, panoramic radiographic evaluation is not included in IMWG protocols for a complete radiographic investigation status in MM patients (2,3,26,27).

In conclusion, the present study suggests that panoramic radiograph may aid to the radiographic protocols to identify MM bone lesions, since radiographic techniques for jawbones and skull (frontal and lateral) were potentially equally able to detect punched-out lesions in each corresponding topography. The benefit for using digital panoramic radiography is the specific identification of punched-out lesions on the jawbones by a simple tool, with low cost and low exposure to radiation. Moreover, in view of the wide availability of digital panoramic radiograph, the present study illustrates the contribution that oral assessment can provide early diagnosis, prompt treatment, and prognosis of MM patients.
